# Dimethoxytrityl‐Protected Oligonucleotide Purification With On‐Column Detritylation in Hydrophobic Interaction Chromatography

**DOI:** 10.1002/jssc.70583

**Published:** 2026-11-13

**Authors:** William E. Evans, Leila Bonakdar, Phu Duong, Hidetaka Kobayashi, Jonas Wege, Patrick Endres, Egbert Müller, Romain Dabre, Jukka Kervinen

**Affiliations:** ^1^ Tosoh Bioscience LLC King of Prussia Pennsylvania USA; ^2^ Tosoh Corporation Bioscience Division Shunan Yamaguchi Japan; ^3^ Tosoh Bioscience GmbH Griesheim Germany

**Keywords:** antisense oligonucleotide, depurination, detritylation, gene therapy, hydrophobic interaction chromatography

## Abstract

State‐of‐the‐art techniques using synthetic antisense oligonucleotides and other nucleotide‐based therapeutics have transformed treatment for a wide range of diseases, including neurological, metabolic, cardiovascular, and muscular disorders, many of which were previously incurable. As the biopharmaceutical industry expands into this new therapeutic field, there is increasing demand for robust purification and analytical techniques. Therapeutic oligonucleotides are most often purified by ion pair‐reversed phase (IP‐RP) and anion‐exchange (AEX) chromatography techniques. Here, we present a single‐step chromatography approach utilizing hydrophobic interaction chromatography (HIC) for a 5′‐dimethoxytrityl (5′‐DMT)‐protected 20‐mer single‐stranded DNA oligonucleotide. The HIC process incorporates an optimized on‐column detritylation step at pH 4.0, enabling removal of the 5′‐DMT protective cap from the purified oligonucleotide. This method also effectively eliminated the majority of < 20‐mer oligonucleotide impurities, which were present as deprotected oligonucleotides in the crude starting material. Analytical RP and AEX chromatography methods confirmed nearly complete (99.6%) 5′‐DMT removal and an oligonucleotide recovery rate of 96.9% for the final product. Moreover, an RP chromatographic depurination assay demonstrated that the acidic detritylation procedure did not result in detrimental adenine or guanine purine base loss. The all‐aqueous HIC process is environmentally friendly and avoids the flammability risks associated with RP technique.

## Graphical abstract

Hydrophobic interaction chromatography resin efficiently captures 5′‐DMT‐protected antisense oligonucleotides (ASOs), enabling acid‐induced on‐column detritylation and elution of highly purified ASOs for gene therapy applications.

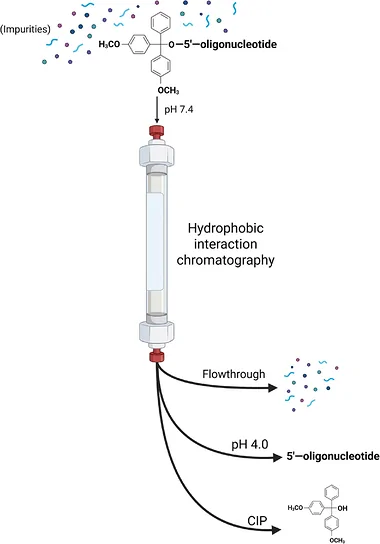

## 1 Introduction

Antisense oligonucleotide (ASO) drugs are short, chemically synthesized single strands of DNA or RNA that interfere with mRNA translation, thus preventing the expression of aberrant proteins [1, 2, 3, 4]. By interacting with RNA transcripts in a sequence‐specific manner, ASOs reduce target RNA transcript levels, thereby limiting or completely halting the encoding of the detrimental proteins. Alternatively, ASOs alter transcript sequences through splice modulation and restore protein function in deficit conditions [5, 6, 7]. Apart from ASOs, nucleic acid strands can also function in a tertiary structure‐dependent manner, including aptamers which utilize protein structural folds that allow them to bind specifically to target sites [3, 8].

ASOs represent a successful broad‐spectrum therapeutic platform that continues to evolve with innovations in the medical field [1, 9, 10, 11]. Promising treatments using ASOs include genetic diseases across neurological, metabolic, cardiovascular, retinal, and muscular conditions, as well as potentiating the efficacy of a hepatitis B vaccine [6, 12, 13, 14, 15, 16]. The rapidly growing interest in ASO therapeutics has necessitated the development of efficient production and analytical methods to limit treatment cost. Oligonucleotide production primarily relies on phosphoramidite‐based solid‐support synthesis, with frequently applied structural modifications designed to improve both potency and stability [17, 18, 19, 20]. Oligonucleotide synthesis is dependent on a protecting group used to temporarily block the 5′‐hydroxyl group of the oligonucleotide. The 5′‐hydroxyl group is typically blocked by an acid‐labile 5′‐DMT (4,4′‐dimethoxytrityl) protective cap to prevent unwanted side reactions. This protective cap is repeatedly added and removed under mildly acidic conditions prior to the addition of the next nucleotide in a cyclical process [19, 21, 22]. The oligonucleotide synthesis process almost invariably leads to a reaction solution mixed with many structurally similar, variable‐length impurities, called shortmers (n−1, n−2, etc.) or longmers (n+1, n+2, etc.) [23, 24]. Due to their molecular similarity to the final product, these impurities are difficult to separate and typically require multiple purification steps.

A variety of liquid chromatography‐based techniques have been developed to remove reaction products and unwanted oligonucleotides produced in the synthesis reactions. The most common purification methods for therapeutic oligonucleotides have been anion‐exchange (AEX) and ion pair‐reversed phase (IP‐RP) liquid chromatography [21, 24, 25, 26, 27, 28, 29, 30, 31]. AEX takes advantage of the negative charge on the oligonucleotide phosphate linkage at alkaline pH to remove the shorter or longer by‐products which are generated during oligonucleotide synthesis. IP‐RP effectively separates oligonucleotides based on their length, sequence, hydrophobic‐protecting groups, and modifications by using ion‐pairing reagents such as alkylammonium salts or other related alkylammonium reagents (e.g. triethylammonium acetate, TEAA). In addition, the polar nature of oligonucleotides makes hydrophilic interaction liquid chromatography (HILIC) a viable alternative for separation and analysis [32, 33, 34, 35]. A recent comprehensive study also describes a hydrophobic interaction chromatography (HIC) process followed by a proposed in‐solution detritylation step for purifying oligonucleotides, demonstrating that it is an all‐aqueous, scalable, and cost‐effective method [36]. Mixed‐mode chromatography, which combines charge and hydrophobicity, has also been successfully applied to oligonucleotide separation [37].

If the crude synthesis mixture contains uncapped oligonucleotides (called “trityl‐off”), it is typically subjected to an IP‐RP chromatography step to remove the newly liberated 5′‐DMT group, followed by AEX chromatography to purify the intact product from the erroneous variable‐length sequences [38]. Alternatively, following oligonucleotide synthesis, 5′‐DMT may also be temporarily left on the full‐length oligonucleotide (called “trityl‐on”) (Figure 1) to provide stability during the subsequent purification process. 5′‐DMT can then later be cleaved in‐solution from the final oligonucleotide product [22, 26]. The authors in [26] also modified the RP process by performing on‐column detritylation under acidic conditions with trifluoroacetic acid (TFA), then eluting the trityl‐off oligonucleotide with an organic solvent. In addition, on‐column detritylation and removal of the protective cap have been described using AEX chromatography [21, 39].

**FIGURE 1:**
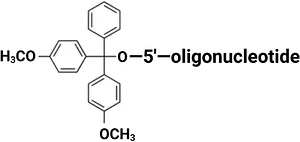
The 4,4′‐dimethoxytrityl (DMT)‐protective cap attached to the 5′‐end of an oligonucleotide.

For oligonucleotides with a DMT‐protective cap attached to the 5′‐end, HIC is also a highly promising purification method due to the strongly hydrophobic 5′‐DMT moiety, as recently demonstrated [36]. Since the chemical bond between 5′‐DMT and the oligonucleotide is labile at low pH (as utilized in the oligonucleotide elongation process [19, 22]), we hypothesized here, similarly to the RP‐based on‐column detritylation process [26], that after binding of the oligonucleotide to the HIC resin, acidification of the mobile phase would cleave the oligonucleotide from the 5′‐DMT, thus releasing it for elution, whereas the DMT cap would remain bound to the resin. The study described here demonstrates an efficient workflow for HIC purification, on‐column detritylation, and analysis of a 20‐mer ssDNA oligonucleotide, supporting ASOs‐targeted therapeutic use.

## 2 Materials and Methods

### 2.1 Chemicals and Reagents

Sodium hydroxide (ACS grade), ammonium sulfate ((NH_4_)_2_SO_4_) (ultrapure), and acetic acid (glacial, ACS grade) were purchased from VWR (Radnor, PA, USA). Uracil (≥ 99.0%), adenine (≥ 99%), guanine (98%), thymine (≥ 99%), cytosine (≥ 99%), LiChrosolv water (LC–MS grade), arginine (ACS grade), triethylammonium acetate (volatile buffer, 0.1 M in water), and ethylenediaminetetraacetic acid (EDTA) were purchased from Sigma–Aldrich (St. Louis, MO, USA). Ammonium formate (99.0%) was purchased from Acros Organics (Geel, Belgium). Ammonium hydroxide was purchased from Ward's Science (Rochester, NY, USA). Acetonitrile (LC/MS grade) was purchased from Supelco (Bellefonte, PA, USA) and acetonitrile (HPLC grade, 99.7+% minimum) from Alfa Aesar (Haverhill, MA, USA). Tris base (ACS grade), sodium phosphate monobasic, and sodium chloride (ACS grade) were purchased from Spectrum Chemical Manufacturing Corporation (New Brunswick, NJ, USA). Water was obtained from a Barnstead E‐Pure ultrapure water purification system from Thermo Scientific (Waltham, MA, USA). The oligonucleotide sample (TriLink BioTechnologies, San Diego, CA, USA) consisted primarily of a 20‐mer ssDNA containing a 5′‐dimethoxytrityl (5′‐DMT) protective cap (Figure 1) and a randomly selected nucleotide sequence of 5′‐GAA TTC ATC GGT TCA GAG AC‐3′, with a reported oligonucleotide purity of 75.3%.

### 2.2 Chromatographic Conditions

HIC was performed with a 0.5 cm I.D. × 5.0 cm length (1.0 mL) column packed with TSKgel Phenyl‐3PW (20) resin (polymethacrylate, particle size 20 µm, pore size 25 nm) (Tosoh Bioscience, King of Prussia, PA, USA). All oligonucleotide purification processes were performed using ÄKTA avant 25 fast performance liquid chromatography (FPLC) instrument (Cytiva, Uppsala, Sweden) at ambient temperature (∼25°C) with in‐line pH, UV, and conductivity detectors. The instrument's U9‐M flow cell had a 2 mm optical path length, a 2 µL cell volume, and an 11 µL total volume. UV absorbance was measured using the default AKTA setting at 254 nm wavelength. The UV sampling rate was variable and automatically adjusted by UNICORN; the UV response time (noise reduction) was 2.5 s, and data acquisition was performed using UNICORN software version 7.3.

For the purification at alkaline pH, the HIC column (1.0 mL) was equilibrated for 10 column volumes (CV) with 10 mM NaOH, 1.0 M (NH_4_)_2_SO_4_, pH 7.4. A flow rate of 0.25 mL/min (75 cm/hour) was used during oligonucleotide loading to achieve a 4 min residence time and was maintained throughout all subsequent purification steps. 5′‐DMT‐containing oligonucleotide (1 mg) sample was adjusted to contain 10 mM NaOH and 1.0 M (NH_4_)_2_SO_4_ prior to loading onto the column. This was followed by column wash with equilibration buffer for 5 CV. Separation was performed in a linearly decreasing (NH_4_)_2_SO_4_ gradient from 1.0 M to 0.05 M in 10 mM NaOH over 10 CV. This was followed by a wash with 10 mM NaOH, 0.05 M (NH_4_)_2_SO_4_ (5 CV), cleaning‐in‐place (CIP) with water (CIP1) for 5 CV, and then with 0.5 M arginine (CIP2) for 5 CV, prior to re‐equilibration.

For the purification with on‐column 5′‐DMT cleavage at pH 4.0, the HIC column (1.0 mL) was equilibrated, sample (0.2 mg) loaded, and the column washed as above. To induce removal of the 5′‐DMT (on‐column detritylation), the column was filled with 0.2 M acetic acid, 1.0 M (NH_4_)_2_SO_4_, pH 4.0, for 2 CV, and then the flow was stopped for variable times. The tested hold times were 0 min, 30 min, 60 min, and 120 min. After restarting the flow, the 5′‐DMT‐free oligonucleotides were collected while the intact 5′‐DMT‐bound oligonucleotides, as well as the cleaved 5′‐DMT cap, remained attached to the column because of 1.0 M (NH_4_)_2_SO_4_. Following this on‐column detritylation, the column was cleaned as described above. For scale‐up experiments, the above procedure was used for a 120 min hold time with 1.0 and 10 mg 5′‐DMT‐oligonucleotide loads, respectively.

### 2.3 Analytical Methods

RP chromatography analyses were performed with a TSKgel OligoDNA‐RP column (0.46 cm I.D. × 15 cm length, silica, particle size 5 µm, pore size 25 nm) (Tosoh Bioscience) using 100 mM triethylammonium acetate, pH 7.0, as the mobile phase A and acetonitrile as the mobile phase B. Runs were carried out using 1.0 mL/min flow rate with a linear gradient from 5% to 35% B over 20 min at 45°C. After the gradient, the column was washed with 80% mobile phase B and re‐equilibrated with 5% mobile phase B. Separation was performed on an Agilent 1100 HPLC (Agilent Technologies, Santa Clara, CA, USA) equipped with a variable wavelength detector (VWD) featuring a 10 mm optical path length and a 14 µL flow‐cell volume. UV absorbance was measured at 254 nm with a sampling rate of 2.5 Hz and a response time of 2.000 s. Data were acquired using ChemStation software, Rev. C.01.08 (210).

AEX chromatography analyses were performed with a TSKgel DNA‐NPR column (0.46 cm I.D. × 7.5 cm length, polymethacrylate, particle size 2.5 µm, non‐porous) (Tosoh Bioscience) using 50 mM Tris‐HCl, 1 mM EDTA, pH 9.0, as the mobile phase A and 50 mM Tris‐HCl, 1 mM EDTA, 1.0 M NaCl, pH 9.0, as the mobile phase B. Runs were carried out using 1.0 mL/min flow rate with a linear gradient from 30% to 55% B over 15 min at 60°C. After the gradient, the column was washed with 100% B for 2 min and re‐equilibrated with 30% mobile phase B. UV absorbance was measured at 254 nm. Separation was performed on an Agilent 1100 HPLC chromatograph with ChemStation software version Rev. C.01.08 (210).

The depurination assay followed the protocol described in [40]. Nucleobase standard stocks were prepared in 0.5 M HCl at 200 µg/mL. Uracil, guanine, and cytosine standards were diluted 1:4 in LiChrosolv water to a concentration of 50 µg/mL each. Adenine and thymine standards were diluted with equal volumes of LiChrosolv water to a concentration of 100 µg/mL. Equal volumes of diluted nucleobase standards were combined to prepare the final nucleobase standards mix. For each run, 5 µL of the standards mix was injected into a column (injected amounts: 50 ng of uracil, 50 ng guanine, 50 ng cytosine, 100 ng of adenine, and 100 ng thymine).

A complete release of guanine and adenine to validate the depurination assay and for quantitation was prepared by spiking the HIC‐purified oligonucleotide sample (from 10 mg oligonucleotide purification lot) with uracil at a ratio of 47 µg oligonucleotide and 4.0 µg uracil and mixing this sample with 50 mM sodium phosphate, pH 1.6, to a final volume of 1.0 mL. The mixture was heated at 90°C for 1 h for the complete release of purines in these harsh conditions [40] prior to analysis. The injected volume was 10 µL. For the analysis of the final oligonucleotide product in the depurination assay, a HIC‐purified oligonucleotide sample (from 10 mg oligonucleotide purification lot) was diluted with LiChrosolv water and mixed with uracil at a ratio of 47 µg oligonucleotide and 4.0 µg uracil (final volume 1.0 mL) prior to analysis. The injected volume was 10 µL.

Depurination detection analyses were performed with a TSKgel ODS‐100 V RP column (0.2 cm I.D. × 15 cm length, silica, particle size 5 µm, pore size 10 nm) (Tosoh Bioscience) using a Thermo UltiMate 3000 U/HPLC instrument (Thermo Fisher Scientific, Waltham, MA, USA) with an autosampler (4°C) for sample holding. The instrument's variable wavelength detector DAD‐3000RS had a 10 mm optical path length and a 13 µL flow‐cell volume. UV absorbance was measured at 254 nm with a sampling rate of 5 Hz and a response time of 1.000 s. Assays were run at 0.25 mL/min flow rate at 40°C. The mobile phase A was 50 mM ammonium formate, pH 7.0, and the mobile phase B was 100% acetonitrile. After sample loading, the linear gradient was from 0% to 4% mobile phase B over 13 min, followed by a wash at 80% B for 3 min. Re‐equilibration was at 0% B for 5 min. Data acquisition was performed using ThermoScientific Chromeleon software, version 7.2.10.

## 3 Results and Discussion

### 3.1 HIC Resin Selection and Purification in (NH_4_)_2_SO_4_ Gradient

HIC exploits the strong affinity between the hydrophobic resin and hydrophobic regions of target molecules. Here, we describe a purification process that utilizes the attached 5′‐DMT group to significantly increase the hydrophobicity of the 20‐mer test oligonucleotide. The addition of 1.0 M (NH_4_)_2_SO_4_ increased the hydrophobic interaction between the resin and the 5′‐DMT‐protected oligonucleotide. We used a phenyl‐substituted HIC modality on a highly crosslinked polymethacrylate base material with 20 µm average particle size and 25 nm pores. The resin's high crosslinking density ensures strong chemical and mechanical stability. We previously also tested other polymethacrylate‐based HIC resins such as PPG‐600 M, Phenyl‐650 M, and Butyl‐650 M, and the data are available in our preliminary report [41]. Nevertheless, Phenyl‐3PW resin was chosen for further characterization due to its most promising performance in elution peak sharpness and resolution, mainly due to its phenyl‐based chemistry and smaller particle size, as opposed to the larger particle size (65 µm) used for the three other HIC resins described above. Using the crude oligonucleotide test material (reported purity 75.3%), we measured a dynamic binding capacity (DBC) of 25 mg/mL for Phenyl‐3PW resin, equivalent to ∼19 mg/mL for the DMT‐capped 20‐mer oligonucleotide (Figure 2A). The initial increase in absorbance from baseline to ∼400 mAU was caused by impurities eluting in the flow‐through fraction.

**FIGURE 2:**
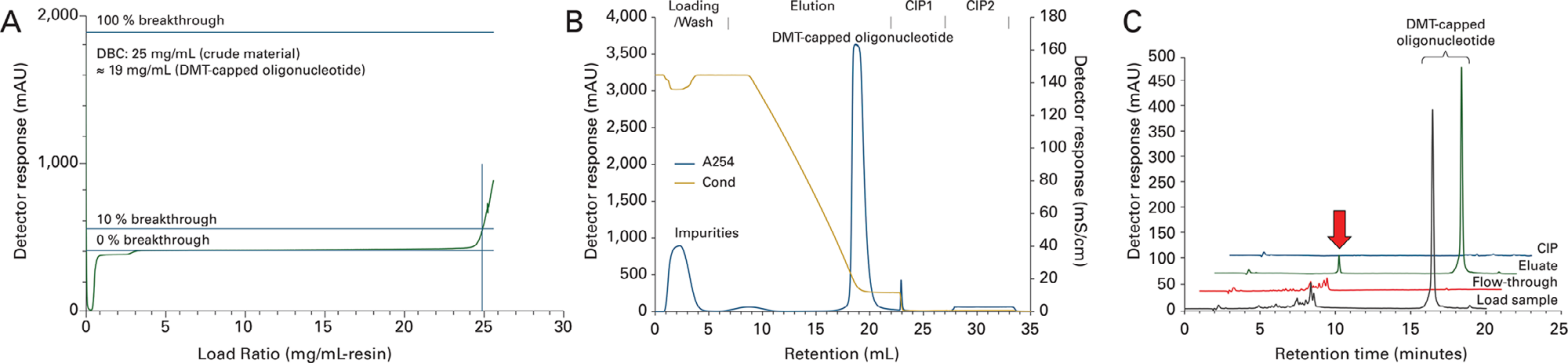
HIC purification of 5′‐DMT‐protected oligonucleotide at pH 7.4. (A) Measurement of the dynamic binding capacity (DBC) of Phenyl‐3PW resin using crude oligonucleotide material. (B) HIC elution profile in a linearly decreasing (NH_4_)_2_SO_4_ gradient from 1.0 M to 0.05 M. (C) RP‐HPLC analysis of the process fractions. The red arrow indicates a small impurity peak, apparently DMT‐free oligonucleotide.

We first tested the 5′‐DMT‐protected oligonucleotide purification in a linearly decreasing (NH_4_)_2_SO_4_ gradient from 1.0 M to 0.05 M (NH_4_)_2_SO_4_ at alkaline conditions (pH 7.4). The run chromatogram (Figure 2B) indicates a loading/wash step with elution of various impurities, and a major oligonucleotide elution peak late in the decreasing salt gradient. RP‐HPLC analysis (Figure 2C) demonstrates a significant improvement in the eluate purity as compared to the load, that can be seen by comparing the load sample, flow‐through, and eluate fractions. Purity of the starting material (75.3%) improved to 94% in the eluate with a recovery of 97%. As anticipated, the oligonucleotide elution peak in the decreasing salt gradient (Figure 2B) contained mainly 5′‐DMT‐protected oligonucleotide, with only a trace of product‐ or process‐related impurities, possibly DMT‐free oligonucleotide (red arrow in Figure 2C). Thus, the 5′‐DMT‐protected oligonucleotide remained largely intact during the purification process if carried out at alkaline pH.

### 3.2 One‐Step HIC Chromatography With Simultaneous On‐Column DMT Cleavage

Removal of the protective acid‐labile 5′‐DMT‐cap (“detritylation”) is typically induced under acidic conditions during every elongation cycle during oligonucleotide synthesis [19, 22]. Similarly, the protective 5′‐DMT‐cap can be cleaved off from the full‐length product in solution at low pH prior to purification. Removal is often achieved by adding trichloro‐ or dichloroacetic acid in dichloromethane solution [22], and the detritylation reaction rate is pH‐ and temperature‐dependent [42]. Following the 5′‐DMT cleavage, the oligonucleotide still requires further polishing to remove the free DMT group as well as other oligonucleotide synthesis by‐products. The purification protocol is typically carried out using either RP‐HPLC or AEX‐chromatography, or combining both RP‐HPLC and AEX methods in the purification process [24, 27, 28, 29].

An alternative approach is to leave the DMT group attached to the 5′‐terminus for protection of the oligonucleotide after synthesis as well as to aid purification since the 5′‐DMT group exhibits a strong affinity to HIC and RP resins, whereas the charged and largely hydrophilic oligonucleotide portion presents reduced (RP) or little (HIC) interaction with these resins. Instead of removing the 5′‐DMT from the oligonucleotide before or between chromatography steps, we modified the HIC purification process to induce on‐column 5′‐DMT cleavage at acidic pH concurrently during purification. Because low pH is also known to strongly promote the harmful depurination reaction [40], we chose relatively mild conditions (0.2 M acetic acid, 1.0 M (NH_4_)_2_SO_4_, pH 4.0, ∼25°C) for our testing. On‐column purification and simultaneous on‐column detritylation were performed using a 1.0 mL HIC column starting with a small 5′‐DMT‐oligonucleotide load (0.2 mg). Sample material, as well as mobile phases for equilibration, wash, and pH 4.0 elution, contained 1.0 M (NH_4_)_2_SO_4_ to hold the 5′‐DMT cap fully attached to the HIC resin.

To promote and maximize on‐column 5′‐DMT cleavage from the oligonucleotide, the optimal hold time at pH 4.0 was investigated in four separate runs. After the loading and washing steps, the column pH was reduced with 2 CVs of pH 4.0 mobile phase to fully saturate the column as seen from the pH trace in Figure 3. The flow was then halted for 0 min, 30 min, 60 min, or 120 min prior to restarting the flow to capture the eluate (Figure 3). The conversion from strongly hydrophobic 5′‐DMT‐capped oligonucleotide starting material to uncapped hydrophilic oligonucleotide with little affinity to the HIC resin was quantified based on the relative FPLC elution peak areas in each chromatogram. Runs with 0 min, 30 min, and 60 min hold times resulted in incomplete 5′‐DMT‐cleavage, and a substantial amount of intact 5′‐DMT‐protected oligonucleotide eluted during the subsequent CIP1 water step at 18–19 min retention time (Figures 3A‐C). In contrast, the 120 min hold time (Figure 3D) was sufficient to convert 93% of the 5′‐DMT‐protected oligonucleotide to the DMT‐free oligonucleotide (Table 1). These proof‐of‐concept experiments showed that efficient 5′‐DMT cleavage and elution of the free oligonucleotide can be performed directly on the column using a minimum hold time of 120 min at pH 4.0 and room temperature.

**FIGURE 3:**
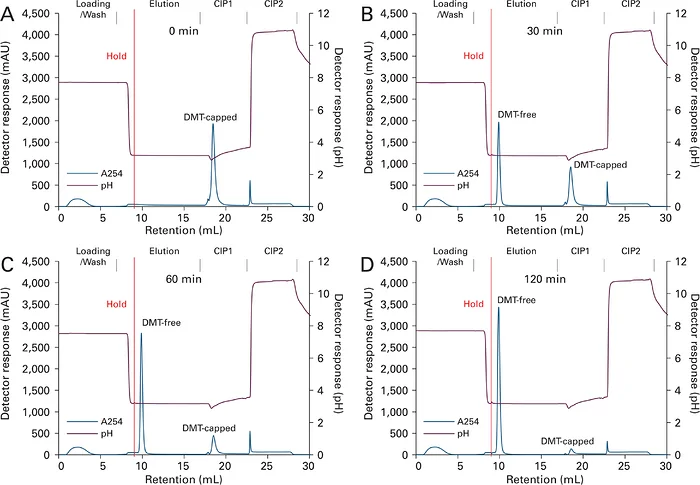
Progressive on‐column 5′‐DMT‐cleavage (detritylation) and oligonucleotide elution at pH 4.0 and after various hold times in four independent runs. The elution peaks of 5′‐DMT‐capped and DMT‐free oligonucleotides are shown after loading 2 CVs of pH 4.0 mobile phase and various hold times of (A) 0 min, (B) 30 min, (C) 60 min, or (D) 120 min. “DMT‐free” indicates elution of oligonucleotides that have the 5′‐DMT‐cleavaged off during pH 4.0 hold. “DMT‐capped” shows elution of oligonucleotides (in CIP1 step with water) with the 5′‐DMT cap attached.

**TABLE 1:** Flow hold times at pH 4.0 and corresponding percent conversion to unprotected (“DMT‐free”) oligonucleotide.

Flow hold time (min)	Conversion to DMT‐free oligonucleotide (%)
0	< 5
30	57
60	78
120	93

### 3.3 Scaling up Sample Load

The oligonucleotide load was incrementally increased from 0.2 mg (Figure 3D) to 1.0 mg (Figure 4A), and subsequently to 10.0 mg (Figure 4B) of 5′‐DMT‐capped oligonucleotide within a 1.0 mL column. A 120 min hold time at pH 4.0 was chosen because it efficiently cleaved 5′‐DMT during scale‐up experiments at room temperature. In Figure 4B, oligonucleotide elution initiated before the hold step started (after 2 CV of pH 4.0 mobile phase) due to high sample load. Fraction collection was triggered at 100 mAU, thus, ensuring the full elution peak was collected.

**FIGURE 4:**
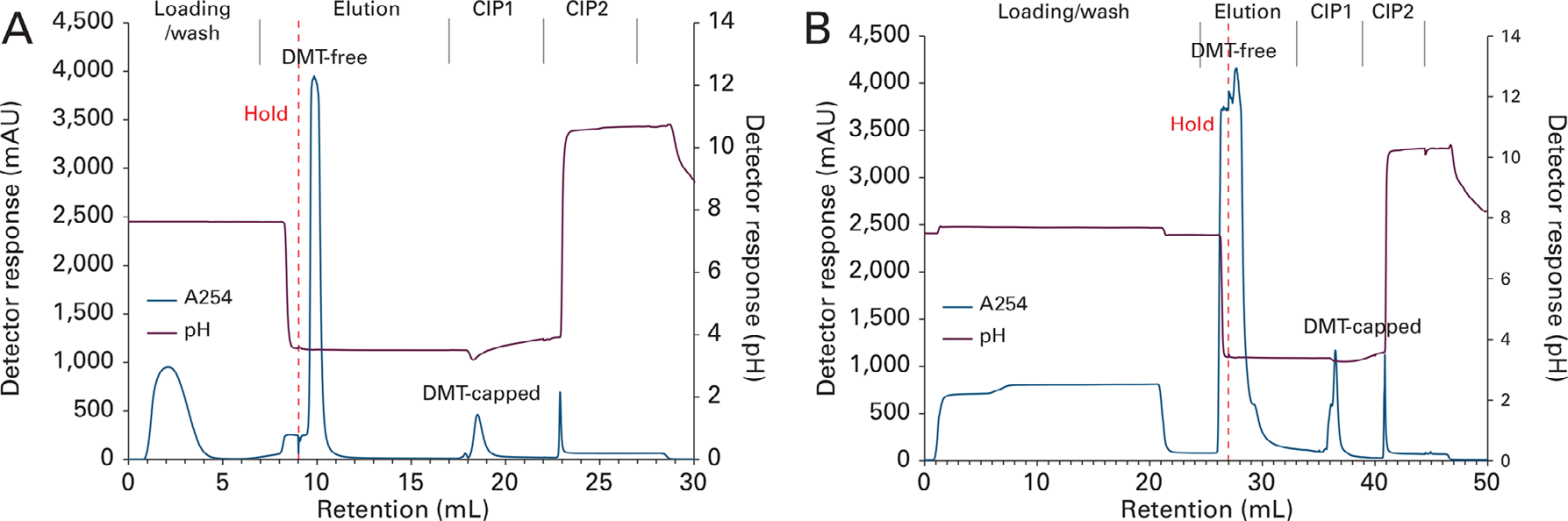
Scaling up oligonucleotide purification in a 1.0 mL HIC column with simultaneous on‐column detritylation at pH 4.0. (A) Elution profile for 1.0 mg 5′‐DMT‐oligonucleotide load and (B) 10.0 mg 5′‐DMT‐oligonucleotide load. Hold time for both samples at pH 4.0 was 120 min prior to elution.

Following each HIC purification, a cleaning‐in‐place protocol was implemented utilizing water and subsequently 0.5 M arginine before re‐equilibration. This approach was selected based on the property of arginine to disrupt hydrophobic interactions in proteins during HIC [43, 44], thereby enhancing the removal of non‐specific impurities from the resin. While not used in this study, an additional sanitization step with 0.5 M NaOH or 20% acetonitrile/methanol can be added as needed to remove hydrophobically bound impurities, followed by thorough water rinsing and re‐equilibration.

### 3.4 Analytical Characterization

#### 3.4.1 On‐Column 5′‐DMT Cleavage Efficiency and Purity Assessment

The oligonucleotide load material that we tested with the HIC column contained a crude mix of intact 5′‐DMT‐capped 20‐mer oligonucleotides, shorter product‐related non‐capped oligonucleotides, and various production‐related impurities. For the purification runs with 1.0 mg and 10.0 mg oligonucleotide loads (Figure 4), the load, flow‐through, and collected fractions were analyzed by RP‐HPLC to determine the extent of DMT removal from the oligonucleotide and the oligonucleotide purity increase during the process. The crude oligonucleotide load contained 76.5% DMT‐protected oligonucleotide (Table 2), as analyzed by RP‐HPLC. Both 1.0 mg and 10.0 mg load amounts achieved > 95% recovery of DMT‐free oligonucleotide, with > 99% oligonucleotide purity, as demonstrated for the 10 mg load sample in Figure 5A and Table 2.

**TABLE 2:** Summary of on‐column 5′‐DMT cleavage efficiency and purity assessment with 10.0 mg oligonucleotide load (1.0 mL column).

Fraction	5'‐DMT‐capped oligonucleotide^a^ (mg)	DMT‐free oligo‐nucleotide^a^ (mg)	Recovery of DMT‐free oligonucleotide^a^ (%)	Oligo‐nucleotide purity^a^, ^c^ (%)	20‐mer oligo‐nucleotide purity^b^ (%)
Load (10 mg)	7.65	2.35	N/A	76.5	70.9
Flow‐through/Wash	Not detected	2.31	N/A	N/A	N/A
Eluate (after pH 4.0 hold, 120 min)	Not detected	7.41	96.9	99.6	90.3
CIP 1	Not detected	0.19	N/A	N/A	N/A
CIP 2	Not detected	0.03	N/A	N/A	N/A

Abbreviations: CIP = cleaning‐in‐place, N/A = not applicable

^a^ RP‐HPLC.

^b^ AEX‐HPLC.

^c^ Contains both 19‐mer and 20‐mer oligonucleotides.

**FIGURE 5:**
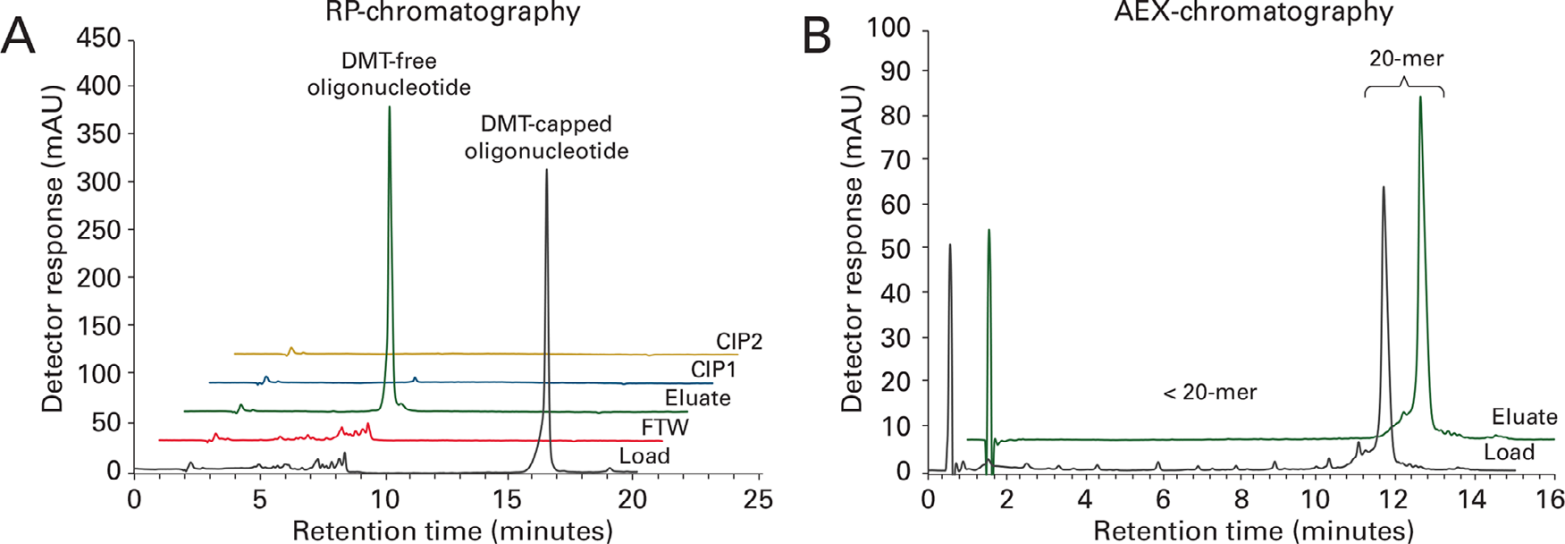
Analysis of elution fractions from the 10.0 mg DMT‐oligonucleotide HIC load. (A) RP‐HPLC elution profiles for the process samples. (B) AEX‐HPLC elution profiles for the load and eluate fractions. Wavelength in detector response was 254 nm for both panels.

AEX‐HPLC analysis (Figure 5B) showed that 70.9% of crude material (“Load”) contained full‐length (20‐mer) oligonucleotide (Table 2); the rest were shortmers (< 20‐mer) of various lengths at retention times from 1 to 11 min. Most shortmers lack the hydrophobic 5′‐DMT protecting group, making them highly hydrophilic. As a result, they were effectively separated from the full‐length 20‐mer oligonucleotide product as early‐eluting product‐related impurities during loading. The eluate sample with simultaneous low pH detritylation during a 120 min flow hold resulted in an almost complete DMT‐free oligonucleotide pool (“Eluate”, Figure 5A). The AEX‐HPLC analysis of the “Eluate” pool indicated that 90.3% (6.69 mg) were full‐length 20‐mer oligonucleotides, the rest being predominantly 19‐mer shortmers (Figure 5B, shoulder on the left side, not fully resolved). The recovery of the DMT‐free oligonucleotide in the eluate fraction was 96.9% (Table 2) based on the peak areas in RP‐HPLC (Figure 5A). It should be noted that the DMT‐protective cap has two absorption maxima at 409 and 510 nm [45], and thus does not have significant absorbance at wavelength 254 nm that would have affected the calculated results based on the peak areas in analytical runs. Furthermore, free DMT was unlikely to coelute with the detritylated oligonucleotide from the HIC column because the pH 4.0 mobile phase contained 1.0 M (NH_4_)_2_SO_4_.

#### 3.4.2 Detection of Possible Depurination

The protective 5′‐DMT cap must be removed from the final oligonucleotide product. However, it is possible that the elongated detritylation step at pH 4.0 may also cause detrimental depurination effects, a type of DNA damage in which N‐glycosidic bonds are cleaved in the oligonucleotide, releasing free purines (adenine and guanine) [40, 46, 47]. Depurination can be quantified using an RP‐HPLC assay [40]. In the assay, the appearance of free adenine and guanine bases are indicative of depurination and can be identified by matching their retention times to the elution profile of the nucleobase standard mix. Accordingly, we prepared a standard mix by combining the four DNA nucleobases: cytosine (C), guanine (G), thymine (T), and adenine (A). In addition, uracil (U) was added as an internal calibration standard to quantify the release of free purines. Then, we separated the nucleobases using RP‐HPLC to determine their retention times. Figure 6A shows the separation of all five nucleobases with clearly distinguishable retention times.

**FIGURE 6:**
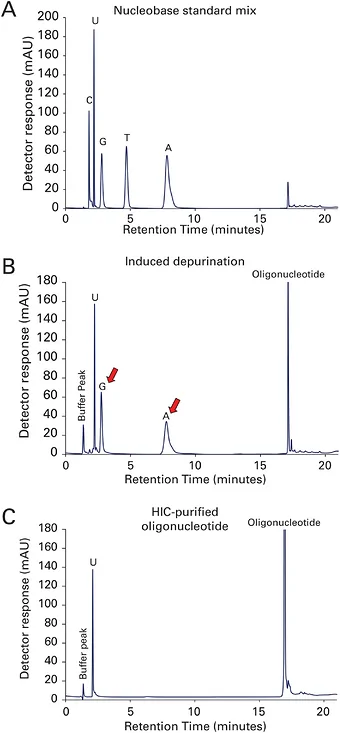
RP‐HPLC analysis of the depurination reaction during HIC purification with on‐column cleavage of 5′‐DMT‐protective cap at pH 4.0. (A) Nucleobase standard mix containing cytosine (C), uracil (U), guanine (G), thymine (T), and adenine (A). (B) The DMT‐free oligonucleotide sample after HIC followed by induced depurination at pH 1.6 with heating (90°C) for 1 hr. (C) Analysis of the DMT‐free oligonucleotide sample after HIC. Uracil (U) was added to oligonucleotide samples as an internal standard. In panel (A), the small peak at the 17‐minute retention time is due to minor oligonucleotide carryover from earlier runs.

To determine the extent of depurination, a complete release of purines from the oligonucleotide was induced under harsh conditions according to [40] as a point of comparison. A sample obtained from the 10.0 mg load run (Figure 4B) was maintained at pH 1.6 for 1 h while heating at 90°C, after which it was subjected to analysis via RP‐HPLC assay. Uracil was spiked into the sample for quantitation of released purines. Figure 6B shows the appearance of guanine and adenine peaks (marked with red arrows), indicating effective depurination. Thus, the RP‐HPLC assay successfully detected depurination as intended. The oligonucleotide elution at a 17 min retention time is also indicated, with a partial peak reaching up to 180 mAU.

To test possible depurination occurring during the HIC‐purification process, the HIC‐purified oligonucleotide sample from the 10.0 mg load run (Figure 4B), uracil was spiked into the sample for quantitation, and the sample was analyzed with the RP‐HPLC assay. Free guanine or adenine was not measurable in the elution chromatogram (Figure 6C), as their amounts were below the limit of detection (LOD), ∼5 ng. Thus, the results demonstrate that the on‐column detritylation process at pH 4.0 did not induce any significant amount (< 5%) of DNA depurination damage to the oligonucleotide.

### 3.5 Comparison of HIC, AEX, and IP‐RP Methods for ASOs

In recent years, there has been tremendous interest regarding the application of diverse oligonucleotides, including ASOs, to treat numerous debilitating chronic illnesses [1, 6, 9, 11, 14]. This has necessitated the development of effective production and purification methods for ASOs and other nucleotide‐based therapeutics. Here, we present a single‐step HIC purification and detritylation method for 5′‐DMT‐protected oligonucleotides that is based on the strongly hydrophobic nature of the 5′‐DMT protective group. The method differs from the conventional AEX and IP‐RP purification methods that are typically used for hydrophilic and charged oligonucleotides after the protective cap has been removed. AEX chromatography resins are particularly suitable for purification of charged oligonucleotides due to their high loading capacity. In the industry, AEX is generally preferred over IP‐RP‐based techniques, which do provide enhanced resolution; however, IP‐RP requires toxic organic solvents that must be effectively removed from the final product to ensure patient safety. When used in large quantities, these solvents also create environmental concerns and pose a flammability risk at the manufacturing site [24, 27, 28, 29, 30, 33]. Like AEX, the HIC method described here does not use toxic organic solvents and provides an additional chromatographic option for oligonucleotide downstream processing.

The oligonucleotide purification method with HIC resin described here was highly effective since, in the presence of 1.0 M (NH_4_)_2_SO_4_, the acid‐induced on‐column 5′‐DMT cleavage from the oligonucleotide effectively separated the column‐bound 5′‐DMT protecting group from the released and collected DMT‐free oligonucleotide pool. In other published methods, the 5′‐DMT group is most often cleaved in‐solution, requiring subsequent desalting or an additional chromatography step to remove it from the purified oligonucleotide product [22, 24]. However, on‐column cleavage of the 5′‐DMT is not a new technique, and previous studies have shown that RP and AEX chromatographic systems can be used successfully for on‐column detritylation. For example, Deshmukh et al. [26] demonstrated the on‐column detritylation and purification of short oligonucleotides by RP and AEX and discussed the potential use of HIC at a larger scale. Their RP method used 1–2% (*v*/*v*) trifluoroacetic acid in water for on‐column detritylation and maintained the depurination reaction below the mass spectrometry detection limit. Neep and Margetts [31] used solid‐phase extraction (SPE) tubes for RP‐based on‐column detritylation and purification, enabling a rapid, high‐throughput process under vacuum. Differences in mobile phases, solvent gradients, and column geometry limit direct comparison with the HIC‐based method described here. Nevertheless, all these approaches demonstrate the suitability of on‐column detritylation within the ASO purification process. Although the HIC‐based on‐column detritylation process described here shares features with other chromatography modes, it offers several advantages: an aqueous solvent system, as used in AEX; early (in flow‐through/wash) removal of mid‐sized (< 20‐mer) failure sequences and small molecules; efficient cleavage and removal of the 5′‐DMT cap from the product; straightforward scalability; and high final oligonucleotide purity. The optimal workflow, either as a single HIC step with on‐column 5′‐DMT detritylation or HIC capture followed by in‐solution 5′‐DMT cleavage and a polishing step, ultimately depends on the oligonucleotide's properties and should be evaluated and optimized individually for each therapeutic ASO.

When the HIC method was used, the only by‐product in the pH 4.0 eluate was a small amount of a 19‐mer oligonucleotide as seen in Figure 5B. This slightly shorter oligonucleotide most probably contains the 5′‐DMT cap, since it binds to the HIC column. Furthermore, it eluted similarly to the 20‐mer oligonucleotide at acidic pH. These n−1 shortmers are often difficult to separate from the full‐length target oligonucleotide [48]. Additional studies will be required to determine the extent of the product‐related 19‐mer impurity and to assess whether its elimination through a robust wash step in HIC, the incorporation of an extra preparative AEX, or other chromatography procedures is necessary for therapeutic purposes. In addition, because the mobile phase used to elute the DMT‐free oligonucleotide at pH 4.0 contains 1.0 M (NH_4_)_2_SO_4_, final formulation of the oligonucleotide product requires a desalting procedure.

### 3.6 Further Refinement of the HIC Process

For improving the HIC method, interesting potential research would be to investigate if the HIC‐based purification methods can reduce other synthesis‐related impurities, such as sulfur substitution (or lack of it) in phosphorothioates leading to diastereoisomers. Moreover, based on our work here and previous studies using RP [26] and AEX [21, 39] chromatography modes, the on‐column detritylation and purification process works effectively for ∼20‐mer 5′‐DMT‐protected oligonucleotides. While not within the scope of this study, investigating the possible effects of nucleotide length or the characteristics of the hydrophobic protective cap on purification efficiency could yield significant knowledge for developing a comprehensive industrial process for an oligonucleotide drug candidate. In addition, further evaluation of 5′‐DMT cleavage below pH 4.0 and at elevated temperatures, while minimizing depurination, may allow the here optimized 120 min hold at ambient temperature to be shortened. However, because both HIC process [36] and susceptibility to depurination [40] depend on multiple factors such as pH, DNA sequence, ionic strength, temperature, and secondary structure (duplex or single‐stranded), optimal conditions should be established separately for each therapeutically relevant oligonucleotide sequence. We hope that the optimized proof‐of‐concept HIC on‐column detritylation approach described herein, together with the supporting analytical methodology, will facilitate broader use of HIC for therapeutic oligonucleotide purification.

## 4 Conclusions

Alongside gene addition and editing, ASOs have become central to genetic medicine, offering disease‐modifying treatments for several otherwise incurable conditions and showing strong future promise [1, 7]. **S**uccessful 5′‐DMT removal and purification of synthetic oligonucleotides is critical for gene therapy applications using ASOs as the therapeutic component. Here, we present an efficient and environmentally sustainable one‐step chromatographic process for 5′‐DMT‐protected 20‐mer ssDNA oligonucleotide using a phenyl‐substituted polymethacrylate HIC resin that includes a simultaneous, on‐column 5′‐DMT cleavage and removal step. Future studies will assess whether this HIC purification method applies to other types of nucleotide‐based biotherapies with hydrophobic caps or linkages designed to improve oligonucleotide stability, cellular uptake, or therapeutic efficiency.

## Author Contributions

**William E. Evans**: conceptualization, investigation, formal analysis, methodology, data curation, validation, visualization, writing – original draft, writing – review and editing. **Leila Bonakdar**: investigation, formal analysis, methodology, data curation, validation, visualization, writing – original draft, writing – review and editing. **Phu Duong**: conceptualization, project administration, writing – review and editing. **Hidetaka Kobayashi**: investigation, formal analysis, project administration, writing – review and editing. **Jonas Wege**: methodology, project administration, writing – review and editing. **Patrick Endress**: methodology, project administration, writing – review and editing, **Egbert Müller**: project administration, Writing – review and editing. **Romain Dabre**: project administration, Writing – review and editing. **Jukka Kervinen**: formal analysis, methodology, data curation, validation, visualization, supervision, project administration, writing – original draft, writing – review and editing.

## Funding

This research was funded by the Tosoh Bioscience LLC. The authors received no specific grants from public, commercial, or not‐for‐profit funding agencies.

## Conflicts of Interest

All authors are employed by Tosoh Corporation, the company that manufactures and markets the HIC process resins and analytical columns described in the study. Aside from this disclosed association, the authors report no other financial or personal interests that could influence the objectivity of this work.

## Data Availability

The data supporting the results and conclusions of this article will be made available on request.
